# Affective and Cognitive Orientations in Intergroup Perception

**DOI:** 10.1177/0146167217699582

**Published:** 2017-04-22

**Authors:** Lukas J. Wolf, Ulrich von Hecker, Gregory R. Maio

**Affiliations:** 1 Cardiff University, UK; 2 University of Bath, UK

**Keywords:** need for affect, need for cognition, stereotypes, intergroup perception, prejudice

## Abstract

Three studies examined the role of need for affect (NFA) and need for cognition (NFC) in intergroup perception. We hypothesized that NFA predicts a preference for stereotypically warm groups over stereotypically cold groups, whereas NFC predicts a preference for stereotypically competent groups over stereotypically incompetent groups. Study 1 supported these hypotheses for attitudes toward stereotypically ambivalent groups, which are stereotyped as high on one of the trait dimensions (e.g., high warmth) and low on the other (e.g., low competence), but not for stereotypically univalent groups, which are seen as high or low on both dimensions. Studies 2 and 3 replicated this pattern for stereotypically ambivalent groups, and yielded provocative evidence regarding several putative mechanisms underlying these associations. Together, these findings help integrate and extend past evidence on attitude-relevant individual differences with research on intergroup perception.

The content of stereotypes differs greatly between groups. For example, whereas Jewish people are seen as successful, strict, and stubborn by Americans, the elderly are seen as dependent and friendly (Cuddy, Fiske, & Glick, 2008; Madon et al., 2001). According to the stereotype content model (SCM; Cuddy et al., 2008; Fiske, Cuddy, Glick, & Xu, 2002), this variability occurs along two dimensions: warmth and competence. Warmth is claimed to be important in interpersonal and intergroup perception because it indicates a person’s or a group’s intention with regard to the self or the ingroup. People and groups that are perceived as good-natured, tolerant, and friendly are seen as benefitting the self and the ingroup, whereas people and groups that are perceived as less tolerant and friendly are seen as harming. Competence plays a role because it indicates the ability to carry out these positive or negative intentions toward the self or the ingroup. Competence subsumes attributes such as intelligence, confidence, and skillfulness. The warmth and competence dimensions are similar to other constructs in the study of person perception, such as agency and communion (Bakan, 1966), self-profitability and other-profitability (Peeters, 2002), or competence and morality (Eagly & Steffen, 1984; Phalet & Poppe, 1997; Wojciszke, Bazinska, & Jaworski, 1998). Abele and Wojciszke (2007) have shown that these constructs overlap to a strong extent.

According to the SCM, another important aspect of this structure is that diverse groups can be organized into clusters, depending on where they are perceived on the warmth and competence dimensions (Cuddy et al., 2008; Fiske et al., 2002). As shown in Figure 1, homeless people and welfare recipients are often seen as relatively low on both warmth and competence (LW/LC cluster), whereas participants’ ingroups and dominant majority groups (e.g., Whites, Christians in the US) are seen as being relatively high on both dimensions (HW/HC cluster). Some groups are evaluated moderately on both dimensions (e.g., Hispanic people, gay men), but many, if not most, social groups are evaluated in an *ambivalent* manner, with relatively low evaluations on one dimension and relatively high evaluations on the other dimension. For instance, whereas groups such as Asian people, rich people, and professionals are stereotyped as higher in competence and lower in warmth (LW/HC cluster), the elderly and people with mental retardation are stereotyped as higher in warmth and lower in competence (HW/LC cluster).

**Figure 1.**
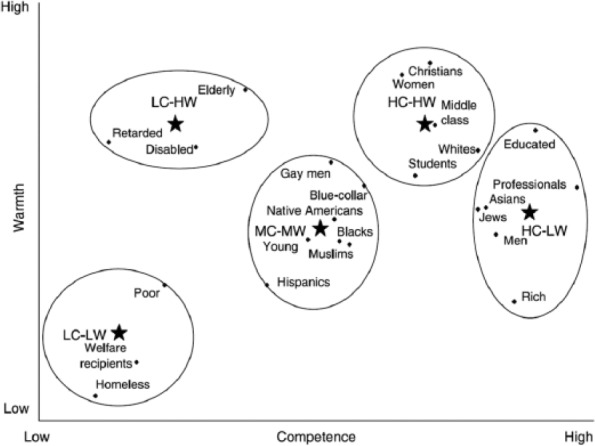
Organization of groups into five clusters along the warmth and competence dimensions. *Source.* Adapted from Fiske et al. (2002). *Note.* HW = higher in warmth; HC = higher in competence; MW = moderate in warmth; MC = moderate in competence; LW = lower in warmth; LC = lower in competence.

An interesting issue is how these stereotypes translate into attitudes toward the groups. According to the SCM, the focus on prejudice as a one-dimensionally negative or positive attitude has obscured the finding that responses can be positive and negative at the same time, depending on the group’s perceived warmth and competence (Fiske et al., 2002). For example, as mentioned above, Asian people are perceived as higher on the competence dimensions and lower on the warmth dimension. Research in interpersonal perception has shown that the relative salience of these dimensions, and hence perhaps the resulting attitude, depends on the situational context and individual differences. For example, Wojciszke (1994) found that participants interpreted ambiguous social behaviors more along the warmth dimension when the behaviors were presented from the observers’ perspective and more along the competence dimension when the behaviors were presented from the actors’ perspective. Moreover, Wojciszke et al. (1998) showed that female participants emphasized the warmth dimension more than male participants.

Overall, this evidence suggests that the situational context and individual differences may play a role in perceptions of groups, with potential implications for the resulting attitude. For instance, a stronger emphasis on stereotypes of Asians as high in competence could result in a more positive attitude toward them than an emphasis on their stereotypically low warmth. The present research builds upon the SCM by proposing that there are important individual differences in how the warmth and competence of groups is evaluated, affecting net attitudes toward the groups.

## Need for Affect (NFA) and Need for Cognition (NFC)

The present research considers two individual difference variables that may be relevant to the warmth and competence dimensions: NFA (Maio & Esses, 2001) and NFC (Cacioppo & Petty, 1982; Cacioppo, Petty, Feinstein, & Jarvis, 1996). NFA has been defined as people’s general motivation to approach or avoid situations and activities that are emotion-inducing for themselves and for others. This includes the desire (or aversion) to experience and understand one’s own and others’ emotions. Consequently, people high in NFA actively seek out and immerse themselves in emotionally evocative stimuli and events. For example, Maio and Esses (2001) found that people high in NFA indicated a greater preference to view emotional films over unemotional films and listed a greater number of emotions, behaviors, and beliefs about a sad emotional event (i.e., Princess Diana’s death) than people low in NFA. Moreover, people higher in NFA have been shown to immerse themselves more readily in a fictional world and give a more positive evaluation of emotions in general (Appel & Richter, 2010; Bartsch, Appel, & Storch, 2010).

NFC has been defined as people’s tendency to engage in and enjoy effortful cognitive activity (Cacioppo & Petty, 1982). For example, Cacioppo and Petty (1982) showed that, after completing a simple and a complex number-circling task, people high in NFC preferred the complex task, whereas people low in NFC preferred the simple task. Similarly, people higher in NFC tended to elaborate more extensively on information provided to them and were more influenced by the strength of arguments (Cacioppo, Petty, & Morris, 1983).

A number of studies have examined NFA and NFC simultaneously. For instance, research has found that NFA more strongly predicts persuasion from persuasive messages that have an affective focus, whereas NFC more strongly predicts persuasion from messages that have a cognitive focus (Haddock, Maio, Arnold, & Huskinson, 2008). These findings indicate that high NFA attunes people to affective information in their environment and that high NFC attunes people to cognitive information in their environment. This difference is interesting in light of the fact that warmth has an affective aspect, because it contrasts traits such as sentimental and humorous with traits such as unsociable and unhappy, whereas competence has a cognitive aspect, because it contrasts traits such as scientific and imaginative with traits such as naïve and unintelligent (Rosenberg, Nelson, & Vivekananthan, 1968). That is, warm targets may be expected to provide more affective stimulation than cold targets, and competent targets may be expected to provide more cognitive stimulation than incompetent targets. Given that NFA predicts liking of affective and emotionally stimulating situations and events (Bartsch et al., 2010; Maio & Esses, 2001), people higher in NFA may favor targets higher in warmth, because they can provide more emotional stimulation. Similarly, given that NFC predicts liking of cognitively challenging situations and events (Cacioppo & Petty, 1982), people higher in NFC may favor targets higher in competence, because they can provide more cognitive challenges.

The present research tests this idea in the intergroup domain, using evidence from the SCM on the positioning of societal groups along the warmth and the competence dimensions. It is noteworthy that this evidence only indicates that groups are high or low in warmth and competence *relative* to the other examined groups. For instance, a stereotypically cold group may not necessarily be perceived as cold per se, but only lower in warmth relative to stereotypically warm groups. Accordingly, we expect that NFA and NFC predict *relative* favorability for some groups compared with others. That is, we expect that higher NFA predicts a preference for groups higher on the warmth dimension over groups lower on the warmth dimension, whereas higher NFC may predict a preference for groups higher on the competence dimension over groups lower on the competence dimension.

A number of relevant considerations are worth noting. First, abundant social-psychological research indicates that various factors are unique to groups as the targets of judgment, including social identity, entitativity, heterogeneity, and permeability (among others). These factors may complicate the study of intergroup attitudes. For instance, Hamilton and Sherman (1996) argued that the main difference between the perception of individuals and groups is that groups are generally perceived as less entitative or coherent, resulting in expectancies and impressions that are formed less easily and spontaneously (Weisz & Jones, 1993). Furthermore, social identity theory (Tajfel & Turner, 1979) posits that individuals who identify more strongly with their ingroup tend to favor their ingroup over relevant outgroups as a means of self-enhancement (Brewer, 1979). Another complexity in intergroup perception is that people may differ in the extent to which they know the common stereotypes of the groups on warmth and competence, endorse the stereotypes, spontaneously activate these stereotypes when encountering the group, and weight the stereotypes in their attitudinal judgment. All these cognitive and motivational factors may weaken the differential impacts of warmth and competence stereotypes on attitudes toward groups.

In addition, an important factor in intergroup perception is people’s desire to be unprejudiced. This individual difference variable is particularly relevant for NFC because people higher in NFC exhibit more socially desirable responding (Cacioppo et al., 1996) and lower explicit prejudice (Waller, 1993). Thus, people higher in NFC might not evaluate groups stereotyped as higher in competence more positively if the desire to appear unprejudiced also inflates the positivity of their attitudes toward groups stereotypically low in competence. Uncertainty in the relative impact of competence weighting and the desire to be unprejudiced makes it difficult to predict the role of NFC a priori.

Overall, then, the psychological jump from interpersonal perceptions to intergroup attitudes is large, and findings on person perception cannot be directly extrapolated to group perception. Nevertheless, prior work on interpersonal perception may provide indirect support for our present research focus (e.g., Hill, 1991). Of particular relevance to the present study, a recent set of experiments by Aquino, Haddock, Maio, Wolf, and Alparone (2016) showed that NFA and NFC predict attitudes at the interpersonal level. In their first experiment, the researchers presented participants with attributes varying along the warmth and competence dimensions. As expected, individuals high in NFA accentuated the difference in valence between warm and cold attributes, whereas individuals high in NFC accentuated the difference in valence between competent and incompetent traits. Furthermore, in the second experiment, participants viewed four fictitious individual targets described as warm, cold, competent, or incompetent, respectively. As expected, people higher in NFA evaluated warm targets more positively than cold targets, but did not show a difference in the evaluation of competent and incompetent targets. Conversely, people higher in NFC evaluated competent targets more positively than incompetent targets, but did not show a difference in evaluation of warm and cold targets. Finally, a third experiment replicated these findings and showed that the evaluation of attributes mediated the associations between NFA and NFC and the evaluation of targets.

These findings for interpersonal attitude provide indirect support for a potential role of NFA and NFC in *intergroup* attitudes, but the SCM highlights another important factor. That is, while Aquino et al. (2016) presented information on *either* warmth or competence for a given individual target, the SCM indicates that groups are usually stereotyped on *both* the warmth and the competence dimensions, and often elicit ambivalent stereotypes (e.g., high in warmth and low in competence). It may be the case that ambivalently stereotyped groups more powerfully differentiate the impact of warmth or competence on attitudes for people high in NFA and NFC, because the ambivalent groups directly oppose the two dimensions. For these groups, NFA and NFC should exhibit an opposing pattern of preferences for the ambivalently stereotyped groups.

In the present three studies, we presented groups that belong to the different clusters identified by the SCM. Study 1 examined how people differing in NFA and NFC evaluated groups from all four clusters of the SCM (i.e., HW/HC, HW/LC, LW/HC, and LW/LC). Studies 2 and 3 focused on groups stereotyped in an ambivalent manner (i.e., HW/LC and LW/HC).

In addition, we examined the putative mechanisms underlying the associations between NFA and NFC and attitudes. First, we looked at the attribute valence mechanism suggested by Aquino et al. (2016): Do individuals higher in NFA favor stereotypically warm groups over stereotypically cold groups because they prefer warm attributes over cold attributes? And do individuals higher in NFC favor stereotypically competent groups over stereotypically incompetent groups because they prefer competent attributes over incompetent attributes? Second, we tested a stereotype content mechanism. That is, NFA and NFC could relate to differences in stereotype content such that participants higher in NFA perceive the groups as differing primarily on warmth, whereas participants higher in NFC perceive the groups as differing primarily on competence. In turn, these accentuations in stereotype content may explain attitudes toward the groups. Third, we tested whether NFA and NFC predicted perceiving the HW/LC groups or the LW/HC groups as more similar to oneself. Additional data from our lab (see supplementary data) indicates that participants higher in NFA perceived themselves higher on warmth and competence. Conversely, participants higher in NFC perceived themselves higher on competence, but not higher on warmth. Hence, people higher in NFA may perceive themselves as more similar to HW/LC groups than to LW/HC groups, whereas people higher in NFC may perceive themselves as more similar to LW/HC groups than to HW/LC groups, and this perceived similarity might account for the differential evaluation of these groups.

## Study 1

Study 1 presented all participants with two HW/HC groups (American people, middle-class people), two LW/LC groups (homeless people, welfare recipients), one HW/LC group (old people), and one LW/HC group (German people).

### Method

#### Participants

We recruited 206 participants online via Prolific Academic (prolific.ac). Six participants failed the Instructional Manipulation Check (IMC; Oppenheimer, Meyvis, & Davidenko, 2009) twice and were excluded from further participation. From the remaining 200 participants (106 men, 94 women; 18-73 years of age, *M*_age_ = 38.13), 156 indicated their ethnicity as European American, 14 as Hispanic American, 11 as Asian American, five as African American, and 14 participants as “Other.” Participants received US$1.25 for their participation in a 10- to 15-min survey.

#### Procedure

First, we presented the IMC to screen out participants who did not read the instructions carefully. In the IMC, text at the top of the screen is followed by a question. However, the text at the top of the screen instructs participants not to answer the question, but instead to confirm that they have read the text. If participants failed to provide the confirmation, they were presented with a warning and the IMC again.

After the IMC, participants completed measures of stereotype content and attitude for each of six groups: two HW/HC groups (American people, middle-class people), two LW/LC groups (homeless people, welfare recipients), one HW/LC group (the elderly), and one LW/HC group (German people), presented in a randomized order. Subsequently, participants completed the NFA and NFC questionnaires and gave warmth and competence valence ratings. Finally, participants indicated the extent to which they perceived themselves as similar to each target group, and they completed social desirability measures before being debriefed.

#### Measures

The stereotype content and attitude measures were identical for each of the six target groups. Here, we describe their application to American people as the target group as an example. Participants were asked to indicate what American people are typically like. Specifically, they were presented with 12 attributes taken from a larger list of 37 attributes (see the appendix), which we adopted from Rosenberg et al. (1968) and Abele and Bruckmüller (2011). Based on a previous study we conducted, we selected the three most highly intercorrelated attributes for each of the four traits: warmth, coldness, competence, and incompetence. The attributes were presented in the following order: ambitious, incompetent, unfriendly, helpful, cold, lazy, skillful, warm, inefficient, competent, good-natured, and boring. Participants rated the extent to which each attribute was characteristic of typical American people on a scale from (1) *very uncharacteristic* to (7) *very characteristic*. After the stereotype content measure, participants indicated their attitude toward American people using a 101-point evaluation thermometer (Haddock, Zanna, & Esses, 1993) from 0° (*extremely unfavorable*) to 100° (*extremely favorable*). We applied the same procedure for the other five target groups.

To measure NFA, we used the short 10-item version by Appel, Gnambs, and Maio (2012). Participants responded to statements such as “I feel that I need to experience strong emotions regularly” on a scale from (1) *totally disagree* to (7) *totally agree*. NFC was measured with the short 18-item version, which comprises such statements as “I find satisfaction in deliberating hard and for long hours” (Cacioppo, Petty, & Kao, 1984). Participants responded on a scale from (1) *extremely uncharacteristic of me* to (5) *extremely characteristic of me*. Both the NFA scale and the NFC scale exhibited good internal consistency (α = .86 and α = .95, respectively; see Table 1 for the descriptive statistics for NFA and NFC).

**Table 1.** Means, Lowest and Highest Scores, Standard Deviation, and Correlation for NFA and NFC in All Three Studies.

	*M*	Minimum	Maximum	*SD*	*r*(NFA/NFC)
Study 1					
NFA	0.82	−2.40	2.80	1.02	.19**
NFC	3.58	1.06	5.00	0.82	
Study 2					
NFA	0.86	−2.90	2.90	1.03	.14*
NFC	3.49	1.22	4.94	0.85	
Study 3					
NFA	0.83	−2.40	2.60	0.93	.31***
NFC	3.71	1.72	5.00	0.77	

*Note.* Possible scores for NFA range from −3 to +3. Possible scores for NFC range from 1 to 5. NFA = need for affect; NFC = need for cognition.

* Significant at .05 level. **Significant at .01 level. ***Significant at .001 level.

For the attribute valence task, we used 24 attributes pertaining to warmth and competence. These attributes were taken from a larger list of 37 attributes (see the appendix), from which we selected the six most highly intercorrelated attributes for each of the four traits based on a previous study in our lab. These 24 attributes were presented in the following random order: humorless, affectionate, determined, naïve, boring, skillful, incompetent, persistent, sociable, unfriendly, lazy, aimless, cold, helpful, happy, inefficient, warm, dismissive, wasteful, intelligent, unpopular, competent, good-natured, and ambitious. Participants were asked to imagine for each attribute that they were meeting people who possessed one of these attributes. Subsequently, they were asked to evaluate these attributes on a scale from (1) *very negative* to (7) *very positive*.

Subsequently, participants indicated their perceived similarity toward each group on a slider from 0 to 100. Next, participants answered the question “how acceptable do you think it is to publicly express negative opinions about American people” on a scale from (1) *completely unacceptable* to (7) *completely acceptable*. Participants answered this question for all six groups. Finally, participants completed a short form of the Marlowe–Crowne social desirability scale (Reynolds, 1982). This scale presented participants with 13 statements such as “No matter who I’m talking to, I’m always a good listener.” Participants indicated for each statement whether it was true or false.

#### Power analysis

In the study by Aquino et al. (2016), NFA and NFC predicted the evaluation of individual targets along the warmth and competence dimension with a medium effect size (β = .30). Based on a recommended power of .80, a power analysis indicated that the required sample size for a medium effect size is 82 participants (G*Power; Faul, Erdfelder, Lang, & Buchner, 2007). Our opportunity samples in all present studies exceeded this requirement.

### Results

Eight participants were excluded because they gave the same response on all thermometer ratings, providing insufficient variability. Moreover, we restricted all analyses to American participants, because the target group “American people” was intended to be participants’ ingroup.^1^ Hence, 184 participants were retained for further analysis. Table 2 shows the descriptive statistics of the favorability ratings toward the groups.

**Table 2.** Descriptive Statistics for Thermometer Ratings Toward the Groups in Study 1.

	*M*	*SD*
HW/HC groups		
American people	71.36	17.26
Middle-class people	73.64	15.56
HW/LC group		
The elderly	69.11	18.51
LW/HC group		
German people	67.76	15.58
LW/LC groups		
Homeless people	45.22	19.54
Welfare recipients	47.97	20.45

*Note.* Possible scores for the thermometer ratings toward groups range from 0 to 100. HW = higher in warmth; HC = higher in competence; LC = lower in competence; LW = lower in warmth.

For the responses on the stereotype content measure, we averaged across attributes for each trait and each type of group. Next, the ratings on the coldness traits were subtracted from the ratings on the warmth traits and the ratings on the incompetence traits were subtracted from the ratings on the competence traits. This created warmth and competence scores for each type of group (all αs > .86).

#### Stereotype content

A repeated measures ANOVA revealed that HW/HC groups and HW/LC groups were perceived as higher on the warmth dimension (*M* = 1.68, *SE* = 0.12, 95% confidence interval [CI] [1.45, 1.91]) than LW/HC groups and LW/LC groups (*M* = 0.36, *SE* = 0.11, 95% CI [0.15, 0.57]), *F*(1, 181) = 116.11, *p* < .001, η^2^_p_ = .39. In contrast, HW/HC groups and LW/HC groups were perceived as higher on the competence dimension (*M* = 2.53, *SE* = 0.11, 95% CI [2.32, 2.74]) than HW/LC groups and LW/LC groups (*M* = −0.26, *SE* = 0.13, 95% CI [−0.52, 0.00]), *F*(1, 181) = 291.80, *p* < .001, η^2^_p_ = .62. Table 3 shows the stereotype ratings for every group on these dimensions and whether these ratings differed significantly from zero. The only negative ratings emerged for LW/LC groups on the competence dimension. Most other groups were perceived positively on both dimensions.

**Table 3.** Descriptive Statistics of Stereotype Content Trait Ratings in Study 1 for Every Dimension and for Every Group.

	*M*	*SE*	*p*	*Cohen’s d*
HW/HC groups				
American people				
Competence	1.64	.14	<.001	0.85
Warmth	1.71	.13	<.001	0.91
Middle-class people				
Competence	2.38	.14	<.001	1.29
Warmth	1.80	.13	<.001	1.02
HW/LC group				
The elderly				
Competence	0.79	.16	<.001	0.37
Warmth	1.60	.15	<.001	0.79
LW/HC group				
German people				
Competence	3.04	.13	<.001	1.71
Warmth	0.59	.16	<.001	0.28
LW/HC groups				
Homeless people				
Competence	−1.50	.17	<.001	0.65
Warmth	−0.08	.14	.55	0.04
Welfare recipients				
Competence	−1.13	.17	<.001	0.48
Warmth	0.34	.15	.024	0.17

*Note.* Possible scores on the warmth and competence dimensions range from −5 to +5. The reported *p* values and Cohen’s *d* indicate the extent to which the scores differ from zero. HW = higher in warmth; HC = higher in competence; LC = lower in competence; LW = lower in warmth.

#### Thermometer ratings

We conducted a repeated measures ANOVA with the factors warmth (warm groups: HW/HC, HW/LC vs. cold groups: LW/HC, LW/LC) and competence (competent groups: HW/HC, LW/HC vs. incompetent groups: HW/LC, LW/LC) and the two continuous variables NFA and NFC. Stereotypically warm groups were evaluated more favorably (*M* = 70.81, *SE* = 1.06, 95% CI [68.72, 72.90]) than stereotypically cold groups (*M* = 57.18, *SE* = 0.93, 95% CI [55.34, 59.01]), *F*(1, 179) = 147.23, *p* < .001, η^2^_p_ =.45. Stereotypically competent groups were evaluated more favorably (*M* = 70.13, *SE* = 0.96, 95% CI [68.24, 72.02]) than stereotypically incompetent groups (*M* = 57.85, *SE* = 1.05, 95% CI [55.79, 59.92]), *F*(1, 179) = 113.94, *p* < .001, η^2^_p_ = .39. Moreover, warmth and competence interacted: stereotypically warm groups were perceived more favorably than cold groups only when they were also stereotyped as low in competence (HW/LC: *M* = 69.11, *SE* = 1.35, 95% CI [66.45, 71.77]; LW/LC: *M* = 46.60, *SE* = 1.34, 95% CI [43.95, 49.25]), *F*(1, 179) = 7.63, *p* = .006, η^2^_p_ = .04, but not when they were stereotyped as high in competence (HW/HC: *M* = 72.50, *SE* = 1.08, 95% CI [70.37, 74.63]; LW/HC: *M* = 67.76, *SE* = 1.15, 95% CI [65.49, 70.03]), *F*(1, 179) = 0.10, *p* = .75, η^2^_p_ = .00.

The continuous variable NFA showed a marginally significant interaction with warmth, *F*(1, 179) = 3.62, *p* = .059, η^2^_p_ = .02: Participants higher in NFA (+1 *SD*) tended to have a stronger preference for stereotypically warm groups over stereotypically cold groups, *M*_diff_ = 15.81, *SE* = 1.61, 95% CI [12.64, 18.98], *F*(1, 179) = 96.95, *p* < .001, η^2^_p_ = .35, than participants lower in NFA (−1 *SD*), *M*_diff_ = 11.45, *SE* = 1.61, 95% CI [8.28, 14.61], *F*(1, 179) = 50.80, *p* < .001, η^2^_p_ = .22. Unexpectedly, NFA also interacted with competence, *F*(1, 179) = 5.48, *p* = .020, η^2^_p_ = .03, showing that participants higher in NFA had a weaker preference for stereotypically competent groups over stereotypically incompetent groups, *M*_diff_ = 14.97, *SE* = 1.64, 95% CI [11.73, 18.22], *F*(1, 179) = 82.90, *p* < .001, η^2^_p_ = .32, than participants lower in NFA, *M*_diff_ = 9.58, *SE* = 1.64, 95% CI [6.34, 12.83], *F*(1, 179) = 33.95, *p* < .001, η^2^_p_ = .16. NFC did not interact with competence, *F*(1, 179) = 0.03, *p* = .86, η^2^_p_ = .00. However, NFC showed a significant interaction with warmth, *F*(1, 179) = 5.26, *p* = .023, η^2^_p_ = .03, such that participants higher in NFC had a weaker preference for stereotypically warm groups over stereotypically cold groups, *M*_diff_ = 16.32, *SE* = 1.61, 95% CI [13.15, 19.48], *F*(1, 179) = 103.24, *p* < .001, η^2^_p_ = .37, than participants lower in NFC, *M*_diff_ = 10.94, *SE* = 1.61, 95% CI [−7.77, 14.11], *F*(1, 179) = 46.42, *p* < .001, η^2^_p_ = .21.

#### Thermometer ratings of ambivalent and univalent groups

**Figure 2.**
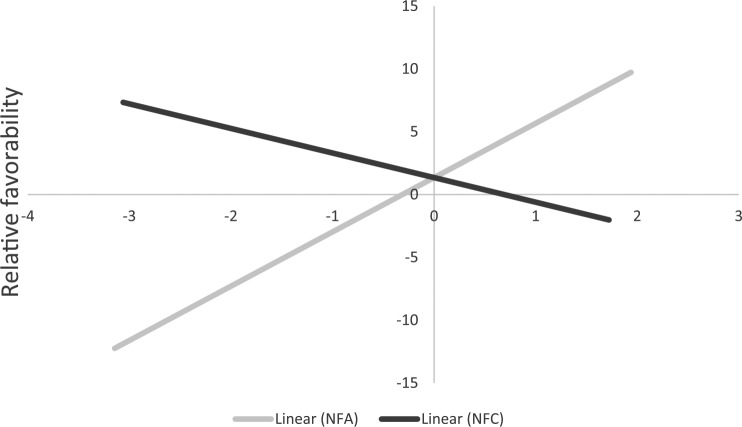
Relative favorability between HW/LC groups and LW/HC groups on standardized NFA and NFC scores in Study 1. *Note.* Higher scores on relative favorability reflect more positivity toward HW/LC groups than LW/HC groups. Possible values on relative favorability range from −100 to +100. NFA = need for affect; NFC = need for cognition; HW = higher in warmth; LC = lower in competence; LW = lower in warmth; HC = higher in competence.

For exploratory purposes, we tested whether the predicted pattern of associations emerged when only considering the ambivalent (i.e., HW/LC and LW/HC) groups. For these analyses, we first subtracted the thermometer ratings toward the LW/HC group from the thermometer ratings toward the HW/LC group and then regressed the relative favorability score onto NFA and NFC. Participants higher in NFA preferred the HW/LC group over the LW/HC group, β = .25, *t*(179) = 3.46, *p* = .001, 95% CI [0.11, 0.40], whereas participants higher in NFC preferred the LW/HC group over the HW/LC group (see Figure 2), β = −.15, *t*(179) = −2.05, *p* = .041, 95% CI [−0.30, −0.01]. In a similar analysis using the univalent (i.e., HW/HC and LW/LC) groups, there were no effects of NFA and NFC, β = −.02, *t*(179) = −0.28, *p* = .78, 95% CI [−0.17, 0.13], β = *−*.10, *t*(179) = −1.34, *p* = .18, 95% CI [−0.25, 0.05]. Table 4 summarizes the findings for all groups.

**Table 4.** Associations Between NFA and NFC and Favorability Ratings in Study 1 for Every Group.

	NFA		NFC	
	β	*p*	β	*p*
HW/HC groups				
American people	.16	.037	−.10	.17
Middle-class people	.14	.059	.00	.96
HW/LC group				
The elderly	.22	.003	−.05	.54
LW/HC group				
German people	−.05	.52	.13	.083
LW/HC groups				
Homeless people	.16	.070	.10	.19
Welfare recipients	.14	.55	.13	.19

*Note.* These associations were obtained by regressing the favorability ratings of the groups on NFA and NFC (simultaneously entered). NFA = need for affect; NFC = need for cognition; HW = higher in warmth; HC = higher in competence; LC = lower in competence; LW = lower in warmth.

#### Social desirability

NFA (*r* = .34, *p* < .001) and NFC (*r* = .24,*p* = .001) were both associated with the social desirability scale, but not with the acceptability ratings aggregated across groups (*r* = −.04, *p* = .59; *r* = −.02, *p* = .77, respectively). Moreover, the social desirability scale only predicted more positive evaluations of the LW/HC groups (*r* = .18, *p* = .015), and the acceptability ratings only predicted lower evaluations of LW/LC groups (*r* = −.30, *p* < .001). In the above analysis of thermometer ratings as an outcome, we also included one of the two social desirability indicators as a further covariate, and in the second step as a moderator of NFA and NFC. Both the acceptability indicator and the social desirability scale did not show a consistent pattern of moderating or suppressing the associations between NFA and NFC and the evaluations of the groups.

#### Mediation analyses

For the associations between NFA and NFC and the relative favorability toward HW/LC groups and LW/HC groups, we tested the mediational roles of attribute valence, stereotype content, and perceived similarity using bootstrapping analyses with 5,000 iterations (Preacher & Hayes, 2008). The first set of analyses tested the simultaneously entered warmth and competence attribute ratings. The warmth attribute ratings were computed by subtracting the average across the cold traits from the average across the warm traits (α = .80). Similarly, the competence attribute ratings were computed by subtracting the average across the incompetent traits from the average across the competent traits (α = .86). The association between NFA and the difference in evaluations of HW/LC groups and LW/HC groups was not mediated by warmth attribute ratings, b = −0.07, *SE* = 0.62, 95% CI [−1.35, 1.11], or by competence attribute ratings, b = −0.04, *SE* = 0.22, 95% CI [−0.70, 0.30]. Similarly, the association between NFC and relative favorability ratings was not mediated by competence attribute ratings, b = −0.03, *SE* = 0.19, 95% CI [−0.66, 0.22], or by warmth attribute ratings, b = 0.00, *SE* = 0.15, 95% CI [−0.27, 0.34].

In a second set of analyses, we replaced the attribute ratings with the simultaneously entered warmth and competence stereotype content ratings. The stereotype warmth score was computed by subtracting the warmth ratings regarding the LW/HC group from the warmth ratings regarding the HW/LC group. Conversely, the stereotype competence score was computed by subtracting the competence ratings regarding the HW/LC group from the competence ratings regarding the LW/HC group. For the association between NFA and relative favorability ratings toward the ambivalently stereotyped groups, stereotype warmth ratings did not function as a mediator, b = 0.18, *SE* = 0.67, 95% CI [−1.10, 1.61]. In contrast, stereotype competence ratings mediated the association, b = 0.98, *SE* = 0.54, 95% CI [0.09, 2.29]. That is, participants higher in NFA perceived a smaller difference between the LW/HC group and the HW/LC group in terms of stereotype competence (path *a*), β = −.16, *t*(179) = −2.18, *p* = .030, 95% CI [−0.80, −0.04], which in turn explained their preference for the HW/LC group over LW/HC group (path *b*), β = −.32, *t*(177) = −5.24, *p* < .001, 95% CI [−0.44, −0.20]. The direct effect remained significant however after including stereotype content ratings (path *c*′), β = .19, *t*(177) = 3.27, *p* = .001, 95% CI [0.08, 0.31]. For the association between NFC and relative favorability ratings, stereotype competence ratings, b = −0.81, *SE* = 0.64, 95% CI [−2.32, 0.26], and stereotype warmth ratings did not function as mediators, b = −0.27, *SE* = 0.88, 95% CI [−2.10, 1.33].

Finally, in a third set of analyses, we replaced the stereotype content ratings with relative similarity ratings. The relative similarity ratings were computed by subtracting the similarity ratings toward the HW/LC group from the similarity ratings toward the LW/HC group. Relative similarity ratings mediated the association between NFC and relative favorability ratings, b = −1.76, *SE* = 0.96, 95% CI [−3.76, −0.01]. That is, participants higher in NFC perceived themselves more similar to the LW/HC group than to the HW/LC group (path *a*), β = −.18, *t*(179) = −2.39, *p* = .018, 95% CI [−0.33, −0.03], and this in turn explained their preference for the LW/HC group over the HW/LC group (path *b*), β = .42, *t*(178) = 6.37, *p* < .001, 95% CI [0.29, 0.55]. The direct effect became nonsignificant when similarity ratings were included in the analysis (path *c*′), β = −.08, *t*(178) = −1.11, *p* = .27, 95% CI [−0.21, 0.06]. In contrast, similarity ratings did not mediate the association between NFA and favorability ratings, b = 0.01, *SE* = 0.77, 95% CI [−1.52, 1.55].

### Discussion

As expected, stereotypically warm groups were perceived as warmer than stereotypically cold groups and stereotypically competent groups were perceived as more competent than stereotypically incompetent groups. Of importance, negative perceptions on the warmth or the competence dimension were virtually absent, restricting the range of influence for these dimensions. This may be part of the reason that Study 1 did not reveal roles for NFA and NFC across all four SCM clusters. Nonetheless, we found support for our hypotheses among the ambivalently stereotyped groups. That is, participants higher in NFA preferred the HW/LC group over the LW/HC group, whereas participants higher in NFC preferred the LW/HC group over the HW/LC group. This finding is consistent with the view that the ambivalent groups test the effects of warmth and competence more strongly by pitting them against each other. In addition, other factors may constrain low favorability ratings for the pure low warmth and low competence groups. However, our analyses of measures of socially desirable responding did not reveal any direct evidence that social desirability is one of these factors and, therefore, other potential explanations merit consideration. For instance, individuals high in NFA may focus on other ways in which LW/LC groups are emotionally engaging (e.g., feeling sympathy), overriding the potentially negative impact of their low warmth. Furthermore, participants low in NFC may be motivated to evaluate their (stereotypically competent) ingroup positively due to social identity motivations, thereby protecting their ingroup. Such processes may restrict our ability to see NFA and NFC associations in line with differences in the groups’ perceived warmth and competence.

Despite this restriction, Study 1 showed that NFA and NFC predicted an opposing pattern of attitudes toward ambivalently stereotyped groups. This interesting pattern is important given the SCM’s finding that most groups are perceived ambivalently (Fiske, Cuddy, & Glick, 2007). Moreover, for these groups, Study 1 provided initial evidence that stereotype competence emerged as a mediator for the relationship between NFA and favorability, while perceived similarity emerged as a mediator for the relationship between NFC and favorability.

## Study 2

Study 1 only included one group in each of the two ambivalent categories. This small sample of groups risks a role for unique stereotypes associated with each group, and therefore potentially a weaker or stronger role for NFA and NFC. In contrast, Study 2 focused on the associations between NFA and NFC and the evaluations of six ambivalently stereotyped groups. In addition, we examined the mediation pattern with this broader range of HW/LC and LW/HC groups.

### Method

#### Participants

We recruited 138 American participants online via Prolific Academic. Thirteen participants failed the IMC (Oppenheimer et al., 2009) twice and were excluded from further participation. From the remaining 125 participants (73 men, 52 women; 18-66 years of age, *M*_age_ = 27.46), 94 indicated their ethnicity as European American, 12 as Asian American, four as African American, three as Hispanic American, two as Middle Eastern, and 10 participants as “Other.” Participants received US$1.96 for their participation in a 15-min survey.

#### Procedure

After passing the IMC, participants completed the same attribute valence task as in Study 1. Subsequently, participants completed thermometer measures of attitude toward the six groups, the NFA (α = .84) and NFC (α = .94) questionnaires (see Table 1 for descriptive statistics), and then similar stereotype content and similarity measures as in Study 1 for the six groups. Study 2 intermixed three HW/LC (housewives, the elderly, and South American people) and LW/HC groups (Asian people, German people, and rich people) in a single fixed order.

#### Measures

To assess participants’ perceived stereotype content, we presented the same 24 attributes as in the attribute valence task in Study 1 and asked participants to rate how much each attribute was characteristic of a typical group member on a 5-point scale from (1) *very uncharacteristic* to (5) *very characteristic.* Apart from these changes, the stereotype content measure was the same as in Study 1, and it was identical for all six groups.

### Results

#### Data preparation

Six participants were excluded because they gave the same response on all thermometer ratings (see Table 5 for descriptive statistics), providing insufficient variability. Moreover, four participants were excluded because they had the same nationality as one of the target groups.^2^ Hence, 115 participants were retained for analysis.

**Table 5.** Descriptive Statistics for Attitude Toward the Groups in Study 2.

	*M*	*SD*
HW/LC groups		
Elderly	65.73	21.01
Housewives	62.33	21.00
South Americans	64.77	18.92
LW/HC groups		
German people	68.88	15.86
Rich people	46.97	19.86
Asian people	69.92	18.31

*Note.* Possible scores for the favorability ratings toward groups range from 0 to 100. HW = higher in warmth; LC = lower in competence; LW = lower in warmth; HC = higher in competence.

As in Study 1, we created a relative favorability score by subtracting the average favorability ratings toward LW/HC groups (α = .69) from the average favorability ratings toward HW/LC groups (α = .68). The responses on the stereotype content measures were aggregated in the same way as in Study 1 (all αs > .88) to create a warmth stereotype content score and a competence stereotype content score for each group type.

#### Manipulation check

Repeated measures *t* tests revealed that HW/LC groups were perceived as higher on the warmth dimension (*M* = 1.26, *SE* = 0.08) and lower on the competence dimension (*M* = 0.60, *SE* = 0.09) than LW/HC groups (*M* = −0.02, *SE* = 0.08; *M* = 1.85, *SE* = 0.10, respectively), *t*(112) = 14.10, *p* < .001, Cohen’s *d* = 1.31, *t*(112) = −10.68, *p* < .001, Cohen’s *d* = −1.00. As shown in Table 6, rich people were perceived as more cold than warm, and Asians were perceived as neutral on the warmth dimension. All other groups were again perceived positively on both dimensions.

**Table 6.** Trait Ratings in Study 2 Averaged for Every Dimension and for Every Group.

	*M*	*SE*	*p*	Cohen’s *d*
HW/LC groups				
Elderly				
Competence	0.31	.11	.007	0.26
Warmth	0.83	.10	<.001	0.75
Housewives				
Competence	0.67	.13	<.001	0.48
Warmth	1.34	.10	<.001	1.24
South American people				
Competence	0.83	.12	<.001	0.64
Warmth	1.63	.12	<.001	1.24
LW/HC groups				
Asian people				
Competence	2.15	.11	<.001	1.86
Warmth	0.17	.12	.18	0.13
German people				
Competence	1.96	.11	<.001	1.60
Warmth	0.25	.10	.017	0.23
Rich people				
Competence	1.43	.13	<.001	1.04
Warmth	−0.47	.11	.001	−0.40

*Note.* Possible scores on the warmth and competence dimensions range from −5 to +5. The reported *p* values and Cohen’s *d* indicate the extent to which the scores differ from zero. HW = higher in warmth; LC = lower in competence; LW = lower in warmth; HC = higher in competence.

#### Thermometer ratings

**Figure 3.**
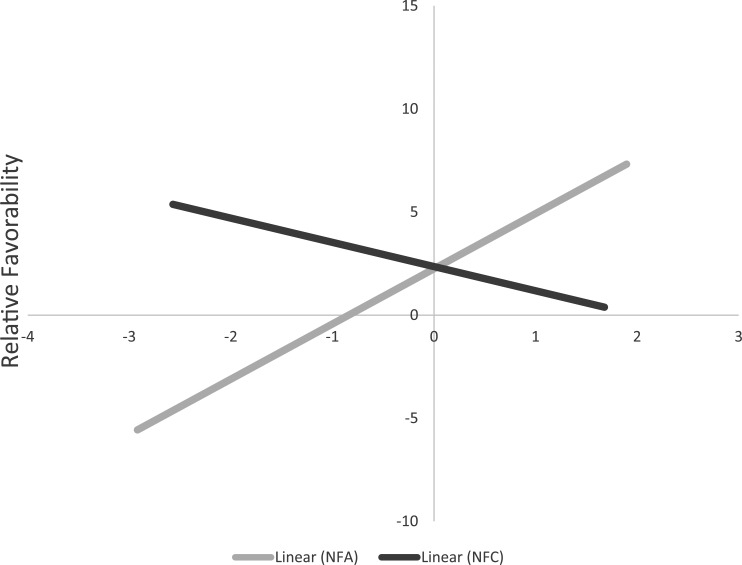
Relative favorability between HW/LC groups and LW/HC groups on standardized NFA and NFC scores in Study 2. *Note.* Higher scores on relative favorability reflect more positivity toward HW/LC groups than LW/HC groups. Possible values on relative favorability range from −100 to +100. NFA = need for affect; NFC = need for cognition; HW = higher in warmth; LC = lower in competence; LW = lower in warmth; HC = higher in competence.

The differences in favorability toward HW/LC groups and LW/HC groups were regressed on NFA and NFC. Consistent with Study 1, participants higher in NFA evaluated HW/LC groups more positively than LW/HC groups, β = .24, *t*(112) = 2.52, *p* = .013, 95% CI [0.05, 0.43], whereas participants higher in NFC tended to evaluate LW/HC groups more favorably than HW/LC groups, β = −.17, *t*(112) = −1.79, *p* = .077, 95% CI [−0.36, 0.02] (see Figure 3). Table 7 presents the associations for each group.

**Table 7.** Associations Between NFA and NFC and Favorability Ratings in Study 2 for Every Group.

	NFA		NFC	
	β	*p*	β	*p*
HW/LC groups				
Elderly	.24	.014	−.14	.16
Housewives	.06	.57	−.15	.12
South American	.22	.023	.10	.32
LW/HC groups				
Asian people	.12	.24	−.01	.96
German people	.04	.68	.19	.051
Rich people	−.06	.53	−.05	.63

*Note.* These associations were obtained by regressing the favorability ratings of the groups on NFA and NFC (simultaneously entered). NFA = need for affect; NFC = need for cognition; HW = higher in warmth; LC = lower in competence; LW = lower in warmth; HC = higher in competence.

#### Mediation analyses

We tested the mediational roles of attribute valence, stereotype content, and perceived similarity in the associations between NFA and NFC and attitudes, using bootstrapping analyses with 5,000 iterations. The first set of analyses tested whether the associations between NFA and NFC and relative favorability ratings were mediated by the simultaneously entered warmth and competence attribute ratings. We aggregated the attribute valence ratings in the same way as in Study 1 to form a warmth attribute score (α = .83) and a competence attribute score (α = .82). The association between NFA and favorability ratings was mediated by warmth attribute ratings, b = 0.29, *SE* = 0.24, 95% CI [0.00, 1.04], but not by competence attribute ratings, b = 0.09, *SE* = 0.17, 95% CI [−0.19, 0.51]. That is, NFA was marginally associated with evaluating warmth more positively (path *a*), β = .17, *t*(112) = 1.78, *p* = .078, 95% CI [−0.02, 0.37], which in turn predicted higher favorability toward HW/LC groups than LW/HC groups (path *b*), β = .26, *t*(110) = 2.36, *p* = .020, 95% CI [0.04, 0.47]. In contrast, NFA did not predict competence ratings, β = −.05, *t*(112) = −0.56, *p* = .57, 95% CI [−0.25, 0.14]. The direct effect became marginally significant when controlling for attribute ratings (path *c*′), β = .18, *t*(110) = 1.90, *p* = .060, 95% CI [−0.01, 0.38].

Conversely, the association between NFC and favorability ratings was mediated by competence attribute ratings, b = −0.94, *SE* = 0.61, 95% CI [−2.66, −0.14], but not by warmth attribute ratings, b = −0.09, *SE* = 0.44, 95% CI [−1.08, 0.76]. In particular, NFC was associated with evaluating competence more positively (path *a*), β = .25, *t*(112) = 2.57, *p* = .012, 95% CI [0.06, 0.44], and the competence attribute ratings in turn predicted higher favorability toward LW/HC groups than HW/LC groups (path *b*), β = −.24, *t*(110) = −2.15, *p* = .034, 95% CI [−0.46, −0.02]. In contrast, NFC did not predict warmth ratings, β = −.02, *t*(112) = −0.22, *p* = .83, 95% CI [−0.22, 0.17]. The direct effect became nonsignificant when controlling for competence attribute ratings (path *c*′), β = −.11, *t*(110) = −1.09, *p* = .28, 95% CI [−0.30, 0.09].

In the second set of analyses, we replaced the attribute ratings with the simultaneously entered warmth and competence stereotype content ratings. These stereotype content scores were aggregated in the same way as in Study 1. The association between NFC and favorability ratings was mediated by stereotype competence, b = −2.48, *SE* = 0.87, 95% CI [−4.62, −1.11], but not by stereotype warmth, b = 0.97, *SE* = 0.67, 95% CI [−0.43, 2.33]. In particular, NFC predicted perceiving LW/HC groups as more competent than HW/LC groups (path *a*), β = .05, *t*(112) = 3.44, *p* = .001, 95% CI [−0.15, 0.24], which in turn explained their preference for LW/HC groups over HW/LC groups (path *b*; see above). The direct effect became nonsignificant when controlling for stereotype content ratings (path *c*′), β = −.08, *t*(110) = −0.90, *p* = .37, 95% CI [−0.25, 0.09]. On the contrary, the association between NFA and favorability ratings was mediated by stereotype competence, b = 1.10, *SE* = 0.40, 95% CI [0.44, 2.07], and not by stereotype warmth, b = −0.51, *SE* = 0.33, 95% CI [−1.23, 0.08]. That is, NFA predicted perceiving less of a difference between LW/HC groups and HW/LC groups in terms of stereotype competence (path *a*), β = −.34, *t*(112) = −3.69, *p* < .001, 95% CI [−0.52, −0.16], which in turn explained their preference for HW/LC groups over LW/HC groups (path *b*), β = −.50, *t*(110) = −5.72, *p* < .001, 95% CI [−0.67, −0.32]. The direct effect became marginally significant when controlling for stereotype content ratings (path *c*′), β = .15, *t*(110) = 1.76, *p* = .37, 95% CI [−0.02, 0.32].

Finally, in a third set of analyses, we replaced the stereotype content ratings with the similarity ratings. As in Study 1, the average similarity ratings for LW/HC groups (α = .53) were subtracted from the average similarity ratings for HW/LC groups (α = .66). These similarity ratings mediated the association between NFC and favorability ratings, b = −1.90, *SE* = 1.02, 95% CI [−4.06, −0.09]. That is, participants higher in NFC perceived themselves more similar to LW/HC groups than to HW/LC groups (path *a*), β = −.21, *t*(112) = −2.15, *p* = .033, 95% CI [−0.40, −0.02], and this in turn explained their preference for LW/HC groups over HW/LC groups (path *b*), β = .57, *t*(111) = 7.41, *p* < .001, 95% CI [0.42, 0.72]. The direct effect became nonsignificant when similarity ratings were included in the analysis (path *c*′), β = −.05, *t*(111) = −0.65, *p* = .51, 95% CI [−0.21, 0.11]. In contrast, similarity ratings did not mediate the association between NFA and favorability ratings, b = 0.63, *SE* = 0.41, 95% CI [−0.12, 1.51].

### Discussion

The results of Study 2 were consistent with the main findings in Study 1: Participants with a higher level of NFA again gave a more favorable evaluation of HW/LC groups than of LW/HC groups, whereas participants with a higher level of NFC tended to give a more favorable evaluation of LW/HC groups than of HW/LC groups. It is significant that the data support a role for these variables despite the restriction in response range (i.e., no groups were seen as strongly low in warmth or competence).

In addition, Study 2 provided further analyses of the underlying mechanism. Evaluations of warmth and competence did not emerge as mediators in Study 1, and the reanalysis of these mediators in Study 2 also showed no support when examining only the groups presented in Study 1 (i.e., the elderly, German people). However, when we examined the entire sample of groups in Study 2, we found evidence that associations with NFA and NFC were mediated by evaluations of warmth and competence, respectively. This mediational pattern is consistent with previous findings by Aquino et al. (2016), and Study 2’s findings should be more representative because this study included more groups than Study 1.

Studies 1 and 2 supported the similarity mechanism for NFC, but not for NFA, and both studies showed unexpectedly that the association between NFA and attitudes toward the groups was mediated by a weaker endorsement of the groups’ stereotypical competence. In addition, while Study 2 supported the notion that the relationship between NFC and attitudes was mediated by a stronger endorsement of the groups’ stereotypical competence, this mediation effect was not present in Study 1. These mediational possibilities receive more attention in Study 3.

## Study 3

Study 3 again examined stereotypically ambivalent groups, while including a different set of groups than in the prior studies. Moreover, Study 3 helped to examine the stereotype content mechanism, which received mixed results across Studies 1 and 2.

### Method

#### Participants

Two samples were selected from different online recruitment websites. For Sample 1, a total of 120 American participants (68 men, 48 women, four did not report; 22-67 years of age, *M*_age_ = 36.13) were recruited online via Amazon’s Mechanical Turk (mturk.com). All participants successfully completed the IMC (Oppenheimer et al., 2009) within two attempts. Of these participants, there were 100 European Americans, six African Americans, six Hispanic Americans, five Asian Americans, one Middle Easterner, and two who indicated “Other.” Participants received US$1.60 for their participation in a 15-min survey. The second sample consisted of 135 American participants who were recruited online via Prolific Academic. Twelve participants failed the IMC twice and were excluded from further participation. From the remaining 123 participants (61 men, 59 women, three did not report; 18-69 years of age, *M*_age_ = 28.20), participants were 80 European Americans, 19 Asian Americans, seven African Americans, five Hispanic Americans, one of Middle East origin, and 11 who indicated “Other.” Participants received US$1.90 for participating. As described below, we refined the design in the second sample by applying small changes to the measures. However, the two samples yielded similar results and were hence combined in the analyses.

#### Procedure

After completing the IMC, participants in the first sample completed a series of items assessing stereotypes and attitudes for each of six groups, presented in a fixed order that intermixed three HW/LC (children, the elderly, and housewives) and LW/HC groups (Asian people, Jewish people, and German people). The participants in the second sample completed similar stereotype and attitude items for six different groups in a fixed order that intermixed three HW/LC (Italian people, South American people, and Irish people) and LW/HC groups (professionals, feminists, and rich people). Finally, participants completed the NFA (α = .86) and NFC (α = .94) questionnaires (see Table 1 for descriptive statistics) and were debriefed.

#### Measures

In the first sample, the stereotype content measure presented the full set of 37 warmth-related and competence-related attributes (see the appendix). For each group, participants responded to 10 warm attributes, nine competent attributes, nine cold attributes, and nine incompetent attributes, all presented in random order. In the second sample, the stereotype content task presented the same 24 attributes as in Study 2. The response scales for the stereotype content measure and the thermometer measure were the same as in Study 2, and they were identical for all groups in the two samples.^3^

### Results

#### Data preparation

We employed the same exclusion criteria as in Study 2. Nine participants were excluded because they gave identical responses on all thermometer ratings (see Table 8 for descriptive statistics), providing insufficient variability. Moreover, five participants were excluded because they had the same nationality as one of the target groups.^4^ Hence, 229 participants were retained for further analysis.

**Table 8.** Descriptive Statistics for Thermometer Ratings Toward the Groups in Study 3.

	*M*	*SD*
HW/LC groups		
Children	76.18	24.91
Elderly	78.50	19.03
Housewives	79.86	21.41
Italian people	71.89	16.32
South American people	66.78	17.44
Irish people	71.26	17.81
LW/HC groups		
Asian people	73.53	17.69
Jewish people	72.05	21.80
German people	72.65	20.61
Professionals	72.28	16.43
Feminists	47.95	26.44
Rich people	49.80	21.02

*Note.* Possible scores for the thermometer ratings toward groups range from 0 to 100. HW = higher in warmth; LC = lower in competence; LW = lower in warmth; HC = higher in competence.

We subtracted the average favorability ratings toward LW/HC groups (Sample 1: α = .70; Sample 2: α = .13)^5^ from the average favorability ratings toward HW/LC groups (Sample 1: α = .60; Sample 2: α = .77). We aggregated stereotype content ratings as in the previous studies, yielding scores for HW/LC competence, HW/LC warmth, LW/HC competence, and LW/HC warmth (αs>.83).^6^

#### Manipulation check

Repeated measures *t* tests revealed that HW/LC groups were perceived as higher on the warmth dimension (*M* = 1.57, *SE* = 0.06) and lower on the competence dimension (*M* = 0.74, *SE* = 0.06) than LW/HC groups (*M* = 0.22, *SE* = 0.06; *M* = 1.92, *SE* = 0.06, respectively), *t*(228) = 16.06, *p* < .001, Cohen’s *d* = 1.06, *t*(228) = −17.76, *p* < .001, Cohen’s *d* = −1.17. As shown in Table 9, rich people and feminists were perceived as more cold than warm, and children were perceived as more incompetent than competent. All other groups were perceived positively on both dimensions.

**Table 9.** Descriptive Statistics of Trait Ratings in Study 3 for Every Dimension and for Every Group.

	*M*	*SE*	*p*	Cohen’s *d*
HW/LC groups				
Children				
Competence	−0.22	.11	.040	0.20
Warmth	1.41	.08	<.001	1.61
Elderly				
Competence	0.79	.11	<.001	0.70
Warmth	1.13	.10	<.001	1.13
Housewives				
Competence	1.09	.12	<.001	0.84
Warmth	1.69	.10	<.001	1.63
Italian people				
Competence	0.95	.11	<.001	0.82
Warmth	1.86	.10	<.001	1.63
South American people				
Competence	1.00	.11	<.001	0.83
Warmth	1.63	.12	<.001	1.28
Irish people				
Competence	0.78	.10	<.001	0.69
Warmth	1.63	.11	<.001	1.32
LW/HC groups				
Asian people				
Competence	2.07	.10	<.001	2.02
Warmth	0.46	.11	<.001	0.39
German people				
Competence	1.95	.12	<.001	1.62
Warmth	0.48	.13	<.001	0.35
Jewish people				
Competence	1.87	.11	<.001	1.63
Warmth	0.82	.12	<.001	0.66
Professionals				
Competence	2.70	.10	<.001	2.46
Warmth	0.78	.11	<.001	0.66
Feminists				
Competence	1.43	.10	<.001	1.25
Warmth	−0.78	.14	<.001	−0.52
Rich people				
Competence	1.49	.12	<.001	1.09
Warmth	−0.32	.09	.001	−0.31

*Note.* Possible scores on the warmth and competence dimensions range from −5 to +5. The reported *p* values and Cohen’s *d* indicate the extent to which the scores differ from zero.

#### Thermometer ratings

**Figure 4.**
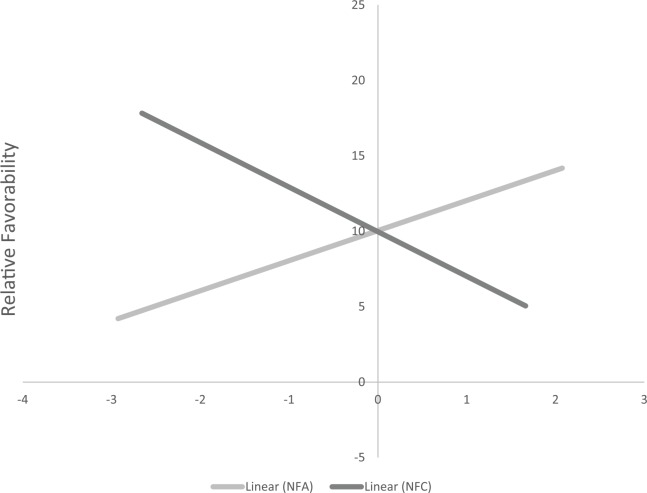
Relative favorability between HW/LC groups and LW/HC groups on standardized NFA and NFC scores in Study 3. *Note.* Higher scores on relative favorability reflect more positivity toward HW/LC groups than LW/HC groups. Possible values on relative favorability range from −100 to +100. NFA = need for affect; NFC = need for cognition; HW = higher in warmth; LC = lower in competence; LW = lower in warmth; HC = higher in competence.

Consistent with Studies 1 and 2, participants higher in NFA evaluated HW/LC groups more positively than LW/HC groups, β = .13, *t*(226) = 1.99, *p* = .048, 95% CI [0.00, 0.26], whereas participants higher in NFC evaluated LW/HC groups more favorably than HW/LC groups, β = −.20, *t*(226) = −3.00, *p* = .003, 95% CI [−0.33, −0.07] (see Figure 4). Table 10 presents the associations for all of the groups.

**Table 10.** Associations Between NFA and NFC and Favorability Ratings in Study 3 for Every Group.

	NFA		NFC	
	β	*p*	β	*p*
HW/LC groups				
Children	.14	.16	.01	.92
Elderly	.23	.018	.03	.76
Housewives	.24	.014	−.06	.50
Italian people	.13	.16	.01	.89
South American people	.16	.080	.06	.56
Irish people	.11	.26	−.02	.81
LW/HC groups				
Asian people	.12	.22	.23	.015
German people	.11	.25	.23	.017
Jewish people	.06	.55	.13	.19
Professionals	−.03	.74	.10	.29
Feminists	.16	.098	.02	.82
Rich people	−.19	.039	.13	.15

*Note.* These associations were obtained by regressing the favorability ratings of the groups on NFA and NFC (simultaneously entered). NFA = need for affect; NFC = need for cognition; HW = higher in warmth; LC = lower in competence; LW = lower in warmth; HC = higher in competence.

#### Mediation analyses

We tested the mediational role of stereotype content in the associations between NFA and NFC and attitudes, using bootstrapping analyses with 5,000 iterations, completed the same way as in the previous studies. The association between NFC and relative favorability ratings was mediated by stereotype competence, b = −0.85, *SE* = 0.41, 95% CI [−1.99, −0.24], but not by stereotype warmth, b = −0.38, *SE* = 0.66, 95% CI [−1.72, 0.89]. In particular, NFC was associated with accentuating the competence dimension (path *a*), β = .21, *t*(226) = 3.16, *p* = .002, 95% CI [0.08, 0.34], which in turn was associated with higher favorability toward LW/HC groups than toward HW/LC groups (path *b*), β = −.21, *t*(224) = −3.62, *p* < .001, 95% CI [−0.32, −0.09]. When the stereotype content score was included in the main analysis, the association between NFC and the difference in favorability became weaker but remained significant (path *c*′), β = −.15, *t*(225) = −2.30, *p* = .022, 95% CI [−0.28, −0.02]. On the other hand, the association between NFA and relative favorability ratings was not mediated by stereotype competence, b = 0.09, *SE* = 0.14, 95% CI [−0.14, −0.44], or stereotype warmth, b = 0.09, *SE* = 0.28, 95% CI [−0.44, 0.66].

### Discussion

The results of Study 3 were consistent with the results of Studies 1 and 2. That is, participants with a higher level of NFA gave a more favorable evaluation of HW/LC groups than of LW/HC groups, whereas people with a higher level of NFC gave a more favorable evaluation of LW/HC groups than of HW/LC groups. Moreover, Study 3 reexamined the stereotype content mechanism. While there was no support for the previous finding that a weaker endorsement of the groups’ stereotypical competence mediated NFA’s effect on attitudes, Study 3 replicated Study 2’s evidence that the effect of NFC on attitudes is mediated by a stronger endorsement of the stereotypical competence of groups.

## General Discussion

The present research investigated the role of NFA and NFC in intergroup perception. Across three studies, the findings revealed that people with a higher level of NFA were more favorable toward groups that are stereotyped as high in warmth and low in competence than toward groups that are stereotyped as low in warmth and high in competence. Conversely, people with a higher level of NFC were more favorable toward groups that are stereotyped as high in competence and low in warmth than toward groups that are stereotyped as low in competence and high in warmth. Studies 2 and 3 consistently obtained this pattern across a range of ambivalently stereotyped groups, thus demonstrating the robustness of our findings.

**Figure 5.**
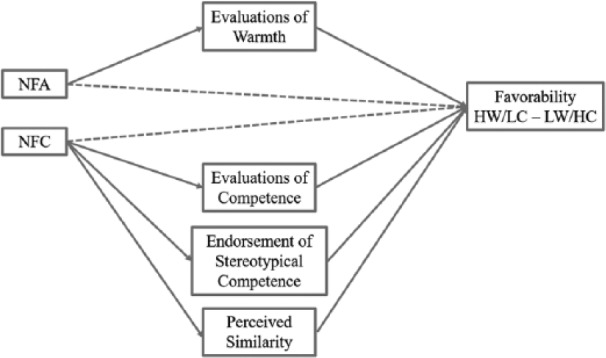
Supported mechanisms across all three studies. *Note.* Across all three studies, these depicted mechanisms received most support in explaining associations between NFA and NFC and favorability toward HW/LC versus LW/HC groups. The effect of NFA was mediated by more positive evaluations of warmth. The effect of NFC on favorability was mediated by more positive evaluations of competence, stronger endorsement of stereotypical competence of the groups, and higher perceived similarity with the groups. NFA = need for affect; NFC = need for cognition; HW = higher in warmth; LC = lower in competence; LW = lower in warmth; HC = higher in competence.

Each study also examined the putative mechanisms underlying these associations. Figure 5 shows the mechanisms that received the most support across all three studies. One potential mechanism entailed evaluations of warmth and competence as mediators. Although Study 1 found no initial support for this mechanism, Study 2’s larger sample of groups showed that the effects of NFA and NFC on attitudes are attributable to differences in the evaluations of warmth and competence, consistent with previous research by Aquino et al. (2016). Hence, there is provocative evidence that NFA predicts favoring high warmth over low warmth, which in turn explains the preference for stereotypically warm but incompetent groups. Conversely, NFC predicts favoring high competence over low competence, which in turn explains the preference for stereotypically cold but competent groups.

Another potential mechanism concerned the endorsement of relevant stereotype content as a mediator. While Studies 1 and 2 provided some evidence that the relationship between NFA and attitudes was mediated by a weaker endorsement of the groups’ stereotypical competence, this mediation effect was not replicated in Study 3’s large sample of groups. Thus, there was no consistent support for this mediational role of stereotype competence in NFA’s effect on attitudes. However, as expected, Studies 2 and 3 showed that the effect of NFC on attitudes was mediated by a stronger endorsement of the groups’ stereotypical competence. Although this mediation was not present in Study 1’s small sample of groups, the more representative samples of groups in Studies 2 and 3 increase our confidence that this mechanism is viable.

Finally, the results of Studies 1 and 2 indicated that perceived similarity of the group to the self also plays a mediating role for people higher in NFC, but not for people higher in NFA. That is, people higher in NFC perceive themselves as more similar to stereotypically cold and competent groups than to stereotypically warm and incompetent groups, which in turn is associated with their preference for the former type of groups over the latter. This is consistent with additional data from our lab showing that people higher in NFC perceive themselves to be more competent, but not warmer (see supplementary data).

The present studies also illustrate some potential boundary conditions for the obtained findings. For instance, all three studies revealed a virtual absence of negative associations and perceptions of the groups. Surprisingly, however, we did not find any evidence that social desirability played a role in this positivity. Nevertheless, the positivity may also be elicited by social identity concerns or other salient attributes of the groups that are weighted more heavily in people’s attitudes toward the groups. These factors may be especially likely to influence results for groups high or low in both warmth and competence given that these are generally participants’ ingroups or strongly negative outgroups.

These complexities in the study of stereotypically univalent groups are one reason why it made sense to focus on ambivalent groups. Another reason was that research on the SCM suggests that most groups are perceived ambivalently (Fiske et al., 2007). A third reason is that, theoretically, the ambivalent groups place NFA and NFC more strongly into conflict because of the manner in which they promote each trait dimension in opposition. Indeed, Study 1 revealed effects of NFA and NFC on these groups but not on the univalent groups, and all three studies revealed consistent evidence that NFA and NFC predicted opposing preferences for the two types of stereotypically ambivalent groups.

Nevertheless, it would be useful to learn more about individual differences in the evaluation of stereotypically univalent groups and to examine factors such as social identity in more detail. Hence, future research could benefit from including a measure of identification with the ingroup. Additional data from our lab suggest that NFA and NFC do not predict evaluations of fictitious groups that are described as high or low in warmth and competence (see supplementary data). However, given that fictitious groups may provide a means to circumvent some biasing factors such as social identity, future research could test under which conditions fictitious groups do elicit effects.

Moreover, on a different note, future research could test whether it may also be fruitful to utilize other measures of attitudes toward the various groups and examine their associations with NFA and NFC. We used a version of the thermometer that is strongly related to both affective and cognitive components in the measurement of intergroup attitudes (Eagly, Mladinic, & Otto, 1994; Esses, Haddock, & Zanna, 1993; Haddock et al., 1993), suggesting that our measure did not favor affective or cognitive processes per se. Another interesting issue is whether implicit measures of intergroup attitude would reveal similar effects. Implicit measures vary a great deal in their measurement characteristics and may tap spontaneous associations that are more affective or cognitive in nature. Hence, it may be useful to examine the role of NFA and NFC using such alternative measures.

Finally, it is worth noting that this research considered the contributions of NFA and NFC to the evaluation of groups independently of each other, that is, the presented analyses did not include the interaction between NFA and NFC. This was the case because no clear predictions could be made for the interaction term, as there is a lack of evidence for NFA × NFC interactions in past research. Nonetheless, in all the present experiments, supplementary analyses examined an alternative model including the interaction term. This model produced no consistent significant findings for the interaction between NFA and NFC. Moreover, the principal conclusions for the separate associations with NFA and NFC across all three studies remained the same and even became stronger in some cases (see supplementary data).

In sum, the present research demonstrates for the first time that NFA and NFC systematically predict attitudes toward ambivalently stereotyped groups. The SCM emphasizes that attitudes toward groups are often ambivalent. The attitudinal impact of this ambivalence depends on individual differences in NFA and NFC, revealing these variables as important factors in the linkage between the SCM and intergroup attitudes.

## Supplementary Material

Supplementary material

Supplementary material

Supplementary material

Supplementary material

Supplementary material

## Appendix

**Appendix:** Stimulus Words Used in Study 1.

Citations	Warm Attributes	Cold Attributes	Competent Attributes	Incompetent Attributes
Rosenberg, Nelson, and Vivekananthan (1968)	Helpful Sentimental Humorous Happy Sociable Good-natured Popular Warm	Vain Finicky Unpopular Boring Humorless Cold	Scientific Determined Persistent Skillful Industrious Intelligent	Wasteful Naïve Submissive Impulsive
Abele and Bruckmüller (2011)	Empathetic Affectionate	Unfriendly Dismissive Reserved	Independent Ambitious Competent	Lazy Insecure Inefficient Aimless Incompetent

## Notes

1. The main conclusions were the same when non-American participants were retained.

2. Results were the same when these participants remained in the analyses.

3. After the stereotype content task, both samples were presented with 24 attributes and asked to select and rank the five most important attributes to understand what a typical target (e.g., child) is like. Aggregating across groups revealed low reliabilities (αs = .07-.71). Consequently, this exploratory measure is not discussed further.

4. The main conclusions were the same when these participants were retained.

5. When the group feminists was excluded in Sample 2, the alpha for the remaining low warmth and high competence (LW/HC) groups (i.e., professionals, rich people) increased to .56. The associations between need for affect (NFA) and need for cognition (NFC) and the evaluation of groups were stronger in this restricted dataset than in the full dataset.

6. These perceived warmth and competence scores were computed across all available attributes in each sample. When excluding the additional attributes present in the first sample, the main conclusions remained the same.
